# Two-year follow-up of revision total hip arthroplasty using a ceramic revision head with a retained well-fixed femoral component: a case series

**DOI:** 10.1186/1752-1947-8-434

**Published:** 2014-12-17

**Authors:** Dirk Ganzer, Lutz Forke, Ulrich Irlenbusch

**Affiliations:** 23 Dietrich-Bonhoeffer-Klinikum, Klosterberg 1a, 17087 Altentreptow, Germany; 24 Marienstift Arnstadt, Orthopädische Klinik, Wachsenburgallee 12, 99310 Arnstadt, Germany

**Keywords:** Ceramic head, Revision, Total hip arthroplasty, Well-fixed stem

## Abstract

**Introduction:**

It is known that a well-fixed stem can be left *in situ* when only the acetabular component and femoral head have to be changed. However, in a revision case, the use of a ceramic head on an existing taper is not recommended. Slight damages of the taper may increase the risk of a ceramic fracture. Until now in a revision case a primary ceramic-on-ceramic or ceramic-on-polyethylene pairing was changed to a metal-on-polyethylene pairing or the well-fixed stem was removed as well. During the past several years, a ceramic head with a metallic sleeve has been introduced as an option for revisions with a stem left *in situ*. We report short-term results of a ceramic revision head in this clinical setting.

**Methods:**

Eight patients with a ceramic revision head were clinically and radiologically followed up two years after revision surgery. Their Harris Hip Score and visual analogue scale scores for pain and satisfaction were recorded, and their radiographs were checked for osteolysis and heterotopic ossifications.

**Results:**

The mean Harris Hip Score increased from 46.5 points before surgery to 88.3 points 2 years after surgery. The mean visual analogue scale score for pain improved from 6.7 to 1.1, and the mean visual analogue scale for satisfaction rose from 5.1 to 8.3. The radiological results did not show osteolysis in any of the patients. Grade I heterotopic ossification according to the Brooker classification system was seen in one patient.

**Conclusions:**

The early clinical and radiological results in this case series are in agreement with previously published studies. Ceramic revision heads with a metallic sleeve are a promising approach in the revision of a ceramic head with a well-fixed stem which can be left *in situ*. This solution avoids an unnecessary exchange of a well-fixed stem and thereby shortens the surgical time of the revision and may reduce the peri-operative complications.

## Introduction

Ceramic material has been used in total hip arthroplasty since 1970 [1]. The two main advantages of ceramic material are its low wear rate and good biocompatibility [2, 3]. During the past 40 years, the properties of ceramic materials have significantly improved, and, as a result, the surgical outcomes have also improved. However, the risk of a ceramic fracture remains because ceramic material is inherently brittle.

It is well known that a precise match of the taper and the femoral head is crucial to avoid stress in localized areas of the ceramic head [4–8]. It is generally not recommended to use a ceramic head on an existing taper during revision surgery, because undetected damage in the taper may increase the risk of ceramic fracture [4, 9–11]. Additionally, a component mismatch may lead to accelerated wear and earlier revision [12]. However, in a revision case, if the femoral stem is well-fixed, it is recommended to change only the femoral head and, if necessary, the acetabular component, while leaving the stem *in situ* [13–15]. Leaving the stem in place reduces blood loss and surgical time [14] and thus peri-operative complication rates [16, 17]. Primary ceramic-on-ceramic or ceramic-on-polyethylene pairings are often revised to metal-on-polyethylene pairings [5, 10, 12, 18].

There are several reasons why a ceramic head is beneficial in revision surgery. Ceramic material is an attractive choice for young and active patients [2, 6, 19] because larger femoral heads can be used, which increases the range of motion and decreases the risk of impingement and dislocation. Bioinert ceramic materials are ideally suited for patients with allergies. Additionally, the revision of a fractured head with a metal-on-polyethylene pairing is not feasible. Severe damage of the metal head and the polyethylene inlay may occur due to third-particle wear, because ceramic particles are much harder than the metal or polyethylene [11, 20].

In recent years, a new option for revision surgery has become available. A ceramic head with an integrated titanium alloy sleeve was developed to allow the use of a ceramic head in combination with a stem left *in situ*. Ceramys^®^ (Mathys Ltd Bettlach, Bettlach, Switzerland) is a nanocrystalline dispersion ceramic material made of a homogeneous dispersion of 20wt% alumina and 80wt% yttrium oxide–stabilized zirconia with a grain size of 0.4μm, a so-called alumina-toughened zirconia. This combination is characterized by a superior resistance against breakage. The wear rate of ceramys® combined with polyethylene is markedly reduced compared to a metal-on-polyethylene pairing [3, 21]. The ceramic head of the model used in this study is fitted with a titanium sleeve. This titanium sleeve allows for the use of the ceramic head without additional revision of a low-damage taper of a stable hip stem. The ceramys® revision head can be combined with a ceramic or polyethylene inlay. Ceramys® revision heads are available with diameters of 28mm, 32mm and 36mm and neck lengths of small (S), medium (M), large (L) and extra-large (XL). In this report, we present the preliminary clinical and radiographic outcomes of a case series of eight patients 2 years after implantation of a ceramic head in revision hip arthroplasty.

## Methods

### Overview

Between December 2007 and October 2008, revision hip arthroplasty using a ceramic revision head was performed in eight patients (Table 1). Inclusion criteria were a revision of the head and cup with a well-fixed stem. The stability of the stem was confirmed by the absence of any radiolucent lines around the stem and no stem migration between the X-ray after index surgery and the X-ray before revision surgery. In addition, the stability was evaluated intra-operatively. No specific exclusion criteria were defined. In all patients, the primary diagnosis was osteoarthritis of the hip joint.

**Figure 1:**
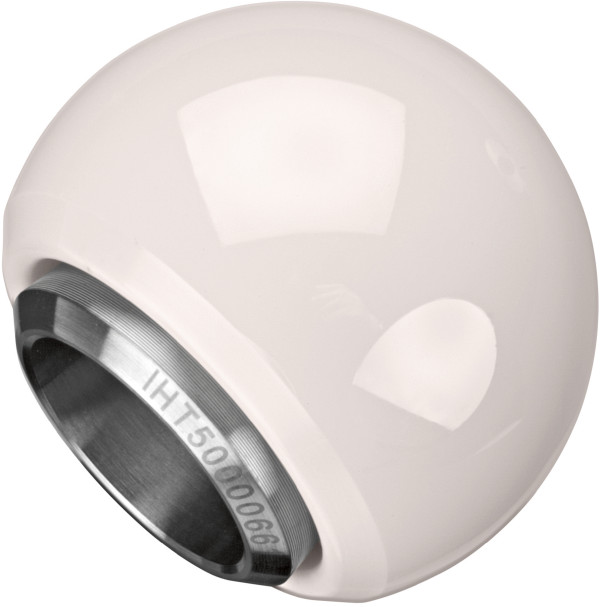
**Ceramys® revision head (permission for use obtained from Mathys).**

The patients were operated on in two clinics in Germany. Surgery was performed using an anterolateral approach. During revision surgery, the cup and head were replaced. Surgery was done according to in-house standards, and patients were followed up by the surgeons in the corresponding clinics. No patient was lost to follow-up, and all patients gave their written informed consent for participation in this study. The authors obtained local Institutional Review Board approval. All patients received a ceramys® revision head with a diameter of 32mm (Figure 1). The mean duration of the revision surgery was 83.4 minutes (range, 60.0 to 157.0 minutes).

**Table 1:** **Demographics**

Characteristics	Values
Gender, *n* (%)	Male, 3 (37.5%); female, 5 (62.5%)
Side operated, *n*	Right, 7; left, 1
Age in yr, mean (range)	65.1 (53.8 to 74.0)
Weight in kg, mean (range)	77.1 (62.0 to 89.0)
Body mass index, kg/m^2^, mean (range)	26.3 (23.0 to 28.7)

Patients were followed up prospectively. The mean follow-up period for the clinical and radiological examinations was 26.7 months (range, 23.0 to 30.4 months). No patient was lost to follow-up.

**Figure 2:**
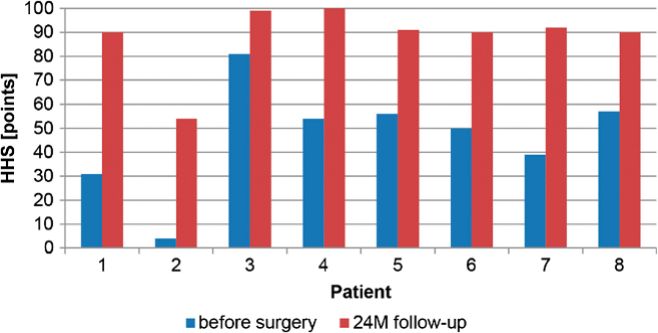
**Harris Hip Score for all patients.** HHS, Harris Hip Score; M, Months.

**Figure 3:**
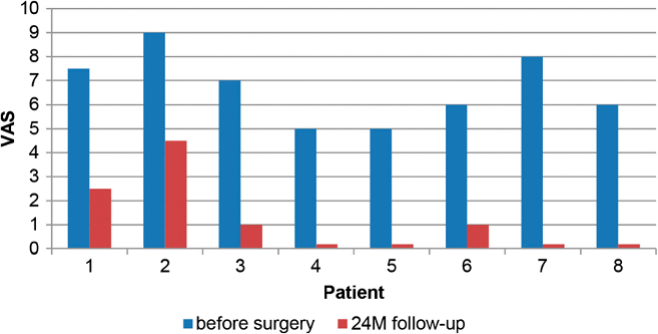
**Visual analogue scale pain scores for all patients.** VAS, Visual analogue scale; M, Months.

**Figure 4:**
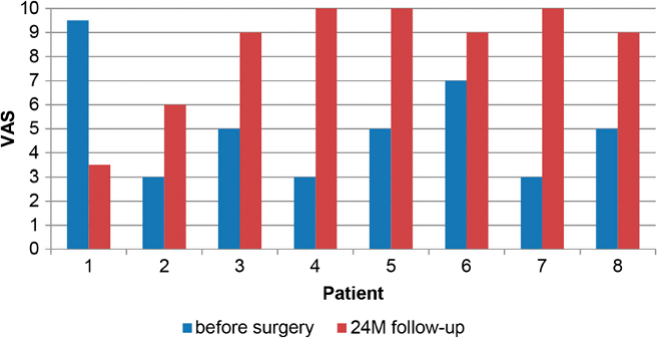
**Visual analogue scale satisfaction scores for all patients.** VAS, Visual analogue scale; M, Months.

The 2-year post-operative radiographs were compared to the first preoperative X-rays and checked for osteolysis, radiolucent lines according to the methods of DeLee and Charnley [22] and Gruen and colleagues [23] and heterotopic ossification according to the Brooker classification [24]. The instances of intra- and post-operative complications were recorded. For the clinical follow-up, the Harris Hip Score (HHS) as modified by Haddad [25] (Figure 2) and the visual analogue scale (VAS) scores for pain (from 0 for no pain to 10 for severe pain) (Figure 3) and patient satisfaction (from 0 for not satisfied to 10 for very satisfied) (Figure 4) were assessed before surgery and at the 2-year follow-up examination.

### Case presentation

#### Case 1

Patient 1 was a 59-year-old Caucasian man who had surgery on the right hip. The paired components in the initial surgery were ceramic-on-ceramic, and the reason for revision was a fracture of the inlay. He received a titanium sleeve size L, and no additional screws were required. His HHS increased from 31 before surgery to 90 at his 2-year follow-up visit. His VAS score for pain improved from 7.5 to 2.5, and his VAS score for satisfaction decreased from 9.5 to 3.5. The VAS satisfaction result of this patient should be treated with caution. Because of impaired mental status, he was not able to objectively report the outcome of the surgery. No heterotopic ossification was observed.

#### Case 2

Patient 2 was a 72-year-old Caucasian man operated on the right hip. The paired components in the initial surgery were ceramic-on–highly cross-linked polyethylene, and the reason for revision was recurrent dislocation. The patient received a titanium sleeve size XL, and no additional screws were required. His HHS increased from 4 before surgery to 54 at his 2-year follow-up examination. His VAS score for pain improved from 9.0 to 4.5, and his VAS score for satisfaction increased from 3.0 to 6.0. No heterotopic ossification was observed.

#### Case 3

Patient 3 was a 73-year-old Caucasian woman operated on the left hip. The paired components in the initial surgery were ceramic-on–highly cross-linked polyethylene, and the reason for revision was wear. The patient received a titanium sleeve size M, and no additional screws were required. Her HHS increased from 81 before surgery to 99 at her 2-year follow-up visit. Her VAS score for pain improved from 7.0 to 1.0, and her VAS score for satisfaction increased from 5.0 to 9.0. No heterotopic ossification was observed.

#### Case 4

Patient 4 was a 73-year-old Caucasian woman operated on the right hip. The paired components in the initial surgery were ceramic-on-polyethylene, and the reason for revision was loosening of the cup. The patient received a titanium sleeve size L, and no additional screws were required. Her HHS increased from 54 before surgery to 100 at the 2-year follow-up examination. Her VAS score for pain improved from 5.0 to 0.0, and her VAS for satisfaction rose from 3.0 to 10.0. No heterotopic ossification was observed.

#### Case 5

Patient 5 was a 53-year-old Caucasian woman operated on the right hip. The paired components in the initial surgery were ceramic-on-polyethylene, and the reason for revision was loosening of the cup. The patient received a titanium sleeve size XL, and two additional screws were required. Her HHS increased from 56 before surgery to 91 at the 2-year follow-up visit. Her VAS score for pain improved from 5.0 to 0.0, and her VAS score for satisfaction went up from 5.0 to 10.0. No heterotopic ossification was observed.

#### Case 6

Patient 6 was a 61-year-old Caucasian man operated on the right hip. The paired components in the initial surgery were ceramic-on-polyethylene, and the reason for revision was loosening of the cup. He received a titanium sleeve size XL, and no additional screws were required. His HHS increased from 50 before surgery to 90 at the 2-year follow-up examination. His VAS score for pain improved from 6.0 to 1.0, and his VAS score for satisfaction rose from 7.0 to 9.0. This patient showed a grade I heterotopic ossification according to the Brooker classification [24].

#### Case 7

Patient 7 was a 62-year-old Caucasian woman operated on the right hip. The paired components in the initial surgery were ceramic-on-polyethylene, and the reason for revision was loosening of the cup. She received a titanium sleeve size XL, and no additional screws were required. Her HHS increased from 39 before surgery to 92 at the 2-year follow-up visit. Her VAS score for pain improved from 8.0 to 0.0, and her VAS score for satisfaction increased from 3.0 to 10. No heterotopic ossification was observed.

#### Case 8

Patient 8 was a 62-year-old Caucasian woman operated on the right hip. The paired components in the initial surgery were ceramic-on-polyethylene, and the reason for revision was loosening of the cup. She received a titanium sleeve size XL, and no additional screws were required. Her HHS increased from 57 before surgery to 90 at the 2-year follow-up examination. Her VAS score for pain improved from 6.0 to 0.0, and her VAS score for satisfaction rose from 5.0 to 9.0. No heterotopic ossification was observed.

## Results

Among all patients, the HHS increased from 46.5 ±22.6 points (mean ± standard deviation) before surgery to 88.3 ±14.4 points at the 2-year follow-up time point. All evaluated patients reported an improvement in the HHS 2 years after surgery (Figure 2). All but one patient (case 2) had a HHS of 90 points or higher at the 2-year follow-up visit.

The VAS score for pain improved from 6.7 ±1.4 to 1.1 ±1.6 (mean ± standard deviation). The values for pain improved for all patients (Figure 3). One-half (50%) of all patients reported that they had no pain 2 years after the surgery.

The VAS for satisfaction improved from 5.1 ±2.3 to 8.3 ±2.3 (mean ± standard deviation), with improvement reported by all but one patient (case 1) (Figure 4). When this patient was excluded from the evaluation because of his impaired mental status, the following were the mean clinical scores after 2 years: VAS pain =0.9 ±1.6, VAS satisfaction =9.0 ±1.4 and HHS =88.0 ±15.6.

**Figure 5:**
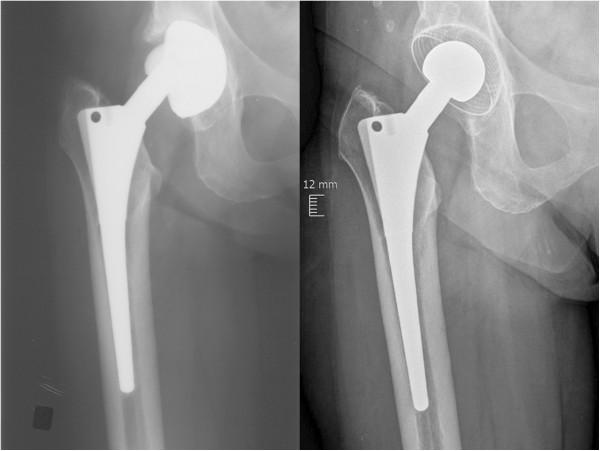
**Representative X-rays taken before revision surgery and at 2 years post-surgery.** The X-ray at left was obtained before revision surgery, and the one at right was taken at 2-year follow-up after revision surgery with a ceramys® revision head combined with an RM Classic Cup and a GSS-CO stem (Mathys Ltd Bettlach, Bettlach, Switzerland).

There was no radiographic evidence of osteolysis or radiolucent lines around the stem or cup for any of the patients after 2 years. No migration of the stem was observed in any of the patients. Representative X-rays taken before revision surgery and at 2 years are shown in Figure 5.

There were no instances of intra-operative complications or complications related to the ceramys® revision head at the 2-year follow-up examination. One patient had a post-operative decubitus ulcer (case 7). None of the patients reported squeaking or other noises related to the implant. None of the patients required a second revision surgery.

## Discussion

There is still no consensus about the best revision surgical strategy in cases where only the acetabular component and femoral head need replacement. It has previously been shown that the stem can remain stable over time when only the cup is replaced [14, 15]. A well-fixed stem should be replaced only under special circumstances where the femoral component is malpositioned, the hip is unstable or a large leg-length difference occurs after revision of the acetabulum. If a monobloc femoral component was used during the initial surgery, the space available for revising the acetabulum will be limited; in these cases, the stem should also be revised [14, 15]. Thus, in most patients, leaving a stable stem is recommended because blood loss and operative time can be reduced and complications related to the exchange of the stem can be minimized [14]. In addition, it is known that peri-operative morbidity, along with other factors, is related to the duration of the surgery [16, 17]. Compared with a revision of both the stem and the cup, the surgical duration in the cases presented here was rather short.

Because of their high resistance to wear and their biocompatibility, ceramic heads are an excellent option for young patients. Ceramic-on-ceramic bearings have relatively low revision rates [2, 26] and even smaller fracture rates [7]. However, cases requiring revision of a ceramic head do arise, and the best revision strategy for these patients is still controversial. Hannouche and colleagues [13] reported implanting standard ceramic heads onto well-fixed stems in 61 revision surgery patients and found no ceramic head fractures after 7 years of follow-up. They concluded that a ceramic head can be placed on an existing taper when no imperfections of the taper are visible [13].

In another report, the 5-year survival rate of the implant after revision due to fracture of the ceramic head was 63% in an overall population of 105 hips [9]. One ceramic head fracture was observed in a patient with a slightly scratched taper. Only 17% of all patients with a ceramic ball head needed a second revision procedure [9]. On the basis of mechanical tests [8] and case reports [4], it is known that even small damage to or contamination of the stem taper decreases the fracture strength of the ceramic head. In addition, a mismatch between the taper and head may lead to accelerated wear and therefore the need for an earlier revision [12]. Thus, a certain risk for fracture or earlier revision exists if small but not insignificant damage of the taper is overlooked and a ceramic head is put on the existing taper.

Specially designed ceramic heads for revision surgery have been available for several years. These revision heads have a metallic sleeve that can be imposed over the taper of a well-fixed stem. The metallic sleeve accommodates minor damage on the existing taper. In that case, a slightly damaged taper can be left *in situ* and a ceramic head can be used without an increased risk for fracture. A limited number of reports of short-term results of revision ceramic heads are available. The outcomes of our study are comparable to those reported by Thorey and co-workers [27], who examined 91 patients after revision surgery in which a revision ceramic head was used (BIOLOX®OPTION; CeramTec, Plochingen, Germany). All of their patients in their study reported an improvement in the HHS and VAS scores. After a mean follow-up time of 2.1 years, the mean HHS was 89.9 points (compared with a mean pre-operative score of 40.0 points), and the VAS score for pain was 1.3 (compared with a pre-operative score of 7.3). These authors concluded that a ceramic revision head is a safe option in a revision case [27]. Their values are similar to our results; in our patients, the 2-year HHS was 88.3 (compared with a pre-operative score of 46.5 points) and the VAS pain score was 1.1 (compared with a pre-operative score of 6.7).

Furthermore, the radiographic outcomes in our study are comparable with those reported in similar studies. Thorey and colleagues [27] observed no signs of component loosening or radiolucent lines 2 years after revision surgery with a ceramic revision head for ceramic fracture, which is in agreement with our present study. Heterotopic ossification was seen in 4 of 91 patients, and all 91 patients were free of symptoms at 2 years [27]. In contrast, Allain and colleagues [9] reported that, after a mean follow-up time of 3.5 years, 21% (*n* =22) of all hips showed radiographic signs of a loosening of the cup and 17% (*n* =3 hips) showed loosening of the stem after revision surgery with a standard ceramic head for a ceramic head fracture. In their multi-center study, different types of prostheses were used.

Because of the relatively rare use of the recently introduced ceramic revision heads, only a small sample size was available for this study and the follow-up time was short, which are clear limitations. The patients will be followed up further to get long-term results, which will be reported when the data are available.

## Conclusions

Our short-term results with the use of the ceramys® ceramic revision head seem promising. The patients’ clinical scores improved, and, from a radiological point of view, no problems arose in the first 2 years after implantation. Thus, a ceramic revision head can be an attractive option for revision cases where the stem is well fixed and can remain *in situ*. The potential for negative side effects of a longer and more complicated revision surgery can be minimized. Owing to the short follow-up time and the very small study group, these results cannot be extrapolated to the general population. Further evaluation of this patient group will be done after longer follow-up.

## Consent

Written informed consent was obtained from the patients for publication of this case report and any accompanying images. A copy of the written consent is available for review by the Editor-in-Chief of this journal.

## Abbreviations

HHS: Harris Hip Score
VAS: Visual analogue scale.
